# Jasmonate-regulated root growth inhibition and root hair elongation

**DOI:** 10.1093/jxb/erac441

**Published:** 2022-11-08

**Authors:** Xiao Han, Mengyi Kui, Kunrong He, Milian Yang, Jiancan Du, Yanjuan Jiang, Yanru Hu

**Affiliations:** CAS Key Laboratory of Tropical Plant Resources and Sustainable Use, Xishuangbanna Tropical Botanical Garden, Chinese Academy of Sciences, Kunming, Yunnan 650223, China; Center of Economic Botany, Core Botanical Gardens, Chinese Academy of Sciences, Kunming, Yunnan 650223, China; CAS Key Laboratory of Tropical Plant Resources and Sustainable Use, Xishuangbanna Tropical Botanical Garden, Chinese Academy of Sciences, Kunming, Yunnan 650223, China; Center of Economic Botany, Core Botanical Gardens, Chinese Academy of Sciences, Kunming, Yunnan 650223, China; University of Chinese Academy of Sciences, Beijing 100049, China; CAS Key Laboratory of Tropical Plant Resources and Sustainable Use, Xishuangbanna Tropical Botanical Garden, Chinese Academy of Sciences, Kunming, Yunnan 650223, China; Center of Economic Botany, Core Botanical Gardens, Chinese Academy of Sciences, Kunming, Yunnan 650223, China; University of Chinese Academy of Sciences, Beijing 100049, China; CAS Key Laboratory of Tropical Plant Resources and Sustainable Use, Xishuangbanna Tropical Botanical Garden, Chinese Academy of Sciences, Kunming, Yunnan 650223, China; Center of Economic Botany, Core Botanical Gardens, Chinese Academy of Sciences, Kunming, Yunnan 650223, China; University of Chinese Academy of Sciences, Beijing 100049, China; CAS Key Laboratory of Tropical Plant Resources and Sustainable Use, Xishuangbanna Tropical Botanical Garden, Chinese Academy of Sciences, Kunming, Yunnan 650223, China; Center of Economic Botany, Core Botanical Gardens, Chinese Academy of Sciences, Kunming, Yunnan 650223, China; CAS Key Laboratory of Tropical Plant Resources and Sustainable Use, Xishuangbanna Tropical Botanical Garden, Chinese Academy of Sciences, Kunming, Yunnan 650223, China; State Key Laboratory for Conservation and Utilization of Bio-Resources in Yunnan, School of Life Sciences, Yunnan University, Kunming, Yunnan 650091, China; CAS Key Laboratory of Tropical Plant Resources and Sustainable Use, Xishuangbanna Tropical Botanical Garden, Chinese Academy of Sciences, Kunming, Yunnan 650223, China; Center of Economic Botany, Core Botanical Gardens, Chinese Academy of Sciences, Kunming, Yunnan 650223, China; Nanjing Normal University, China

**Keywords:** Jasmonate, JAZ proteins, MYC2, RHD6, root growth, root hair elongation

## Abstract

The phytohormone jasmonate is an essential endogenous signal in the regulation of multiple plant processes for environmental adaptation, such as primary root growth inhibition and root hair elongation. Perception of environmental stresses promotes the accumulation of jasmonate, which is sensed by the CORONATINE INSENSITIVE1 (COI1)–JASMONATE ZIM-DOMAIN (JAZ) co-receptor, triggering the degradation of JAZ repressors and induction of transcriptional reprogramming. The basic helix–loop–helix (bHLH) subgroup IIIe transcription factors MYC2, MYC3, and MYC4 are the most extensively characterized JAZ-binding factors and together stimulate jasmonate-signaled primary root growth inhibition. Conversely, the bHLH subgroup IIId transcription factors (i.e. bHLH3 and bHLH17) physically associate with JAZ proteins and suppress jasmonate-induced root growth inhibition. For root hair development, JAZ proteins interact with and inhibit ROOT HAIR DEFECTIVE 6 (RHD6) and RHD6 LIKE1 (RSL1) transcription factors to modulate jasmonate-enhanced root hair elongation. Moreover, jasmonate also interacts with other signaling pathways (such as ethylene and auxin) to regulate primary root growth and/or root hair elongation. Here, we review recent progress into jasmonate-mediated primary root growth and root hair development.

## Teaser

JAZ proteins interact with and suppress bHLH subgroup IIIe and IIId transcription factors to regulate jasmonate-induced root growth inhibition, and repress RHD6 and RSL1 to modulate jasmonate-enhanced root hair elongation.

## Introduction

**Fig. 1.**
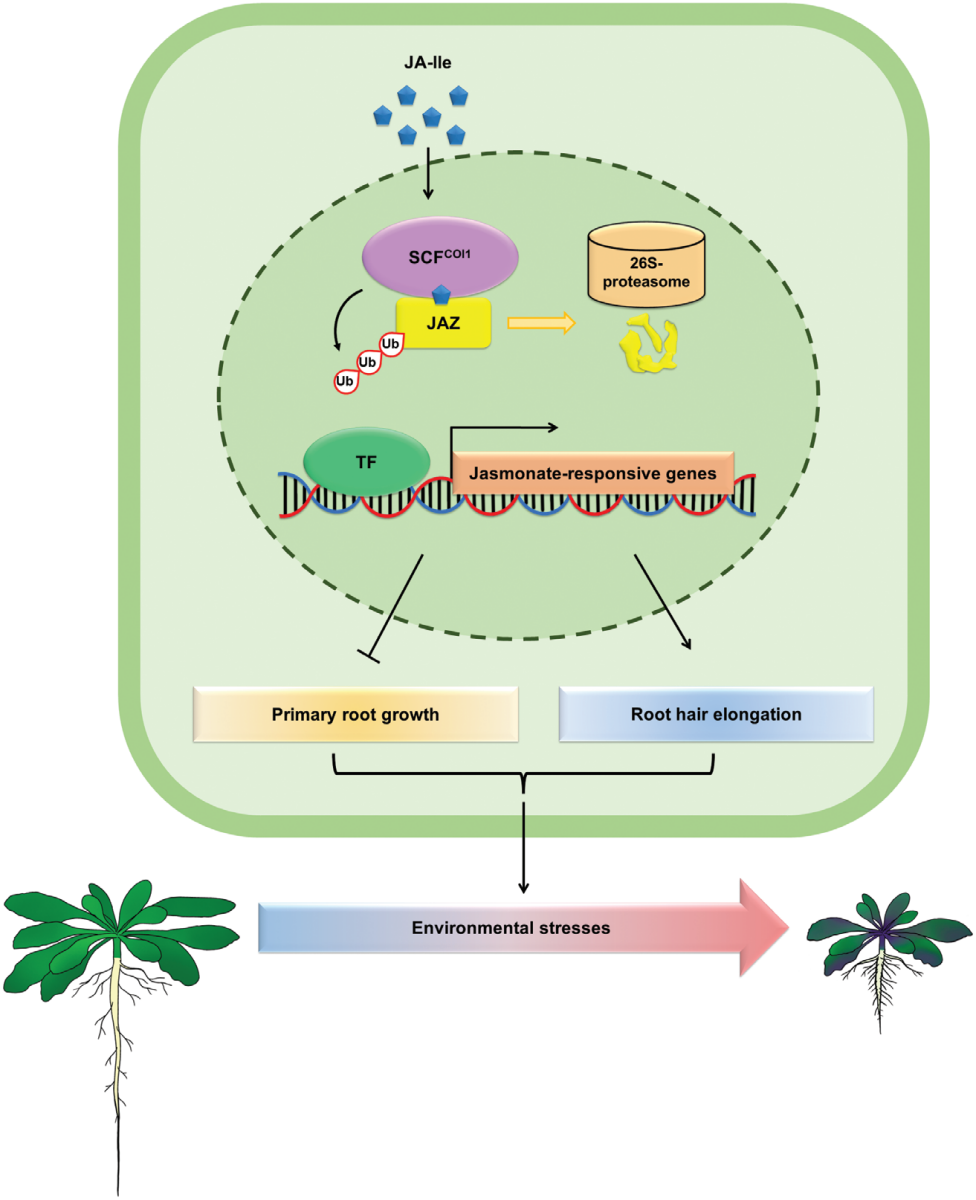
Jasmonate regulates multiple physiological processes for environmental adaptations. The F-box protein CORONATINE INSENSITIVE1 (COI1) forms a co-receptor complex with JASMONATE ZIM-DOMAIN (JAZ) family proteins to perceive jasmonate. After the perception of jasmonate, JAZ repressors are degraded via the SCF^COI1^–26S proteasome pathway. The degradation of JAZ proteins subsequently releases downstream transcription factors to modulate physiological processes. Following environmental stress, jasmonate leads to root growth inhibition and/or root hair elongation, which may lead to increased nutrient acquisition and enhanced stress resistance.

The integration of exogenous factors and endogenous developmental programs, which is required for plants to adapt to adverse environmental conditions, is mediated by complex signaling networks. Specifically, the phytohormone jasmonate is critical for plant adaptation to environmental conditions because it (i) coordinates various physiological processes, such as root growth and anthocyanin accumulation, (ii) enhances the ability of plants to withstand biotic and abiotic stresses, and (iii) balances growth and defense activities to optimize plant fitness in response to limited resources (Chini *et al.*, 2016) (Fig. 1). The F-box protein CORONATINE INSENSITIVE1 (COI1), which is a jasmonate receptor, binds to the Arabidopsis Skp1-like proteins (ASK1/ASK2), the Cdc53-like protein (Cullin1), and small RING finger protein (Rbx1) to form the SCF^COI1^ complex (Xie *et al.*, 1998; Xu *et al.*, 2002; Liu *et al.*, 2004; Ren *et al.*, 2005; Yan *et al.*, 2009). After jasmonate is perceived, JASMONATE ZIM-DOMAIN (JAZ) proteins, which are crucial jasmonate signaling repressors, are degraded via the SCF^COI1^–26S proteasome pathway, resulting in the release of downstream transcription factors to modulate various jasmonate responses (Chini *et al.*, 2007; Thines *et al.*, 2007; Fonseca *et al.*, 2009; Sheard *et al.*, 2010; Qi *et al.*, 2011; S. Song *et al.*, 2011; Hu *et al.*, 2013; Chico *et al.*, 2014; Qi *et al.*, 2014; Jiang *et al.*, 2014; Sethi *et al.*, 2014; Zhai *et al.*, 2015; Chini *et al.*, 2016; Huang *et al.*, 2017; Hu *et al.*, 2017; Han *et al.*, 2018; Guo *et al.*, 2018a; Howe *et al.*, 2018; Wasternack, 2020; Pan *et al.*, 2020; Cao *et al.*, 2022). In this review, we summarize current knowledge concerning the regulatory effects of jasmonate on root growth inhibition and root hair elongation.

## Jasmonate-induced root growth inhibition

### COI1/JAZ-mediated jasmonate signaling suppresses root growth

The F-box protein COI1 is a core positive regulator of jasmonate-dependent responses (Xie *et al.*, 1998; Xu *et al.*, 2002; Yan *et al.*, 2009). The loss-of-function *coi1* mutants are insensitive to jasmonate-induced primary root growth inhibition (Xie *et al.*, 1998; Xu *et al.*, 2002). Additionally, jasmonate promotes stem cell niche regeneration in a COI1-dependent manner. Compared with the wild-type control, in *COI1* mutants following the ablation of the quiescent center, the regeneration responses of a new quiescent center and ablated cells are reportedly delayed (Zhou *et al.*, 2019). In response to stress or endogenous stimuli, bioactive jasmonate induces the interaction between COI1 and JAZ proteins to form a co-receptor complex, leading to the ubiquitination and subsequent degradation of the JAZ repressors (Chini *et al.*, 2007; Thines *et al.*, 2007). In Arabidopsis, the JAZ family comprises 12 canonical members that contain conserved domains (ZIM and Jas) and the atypical JAZ13 repressor with diverse domains (Thireault *et al.*, 2015; Chico *et al.*, 2016; Chini *et al.*, 2016). The overexpression of *JAZ4*, *JAZ8*, *JAZ9*, and *JAZ13* as well as truncated forms or alternatively spliced variants of *JAZ* genes (*JAZ1Δ3A*, *JAZ3ΔC*, *JAZ10.3*, and *JAZ10.4*) results in jasmonate insensitivity, thereby decreasing the inhibitory effects of jasmonate on root growth (Chini *et al.*, 2007; Thines *et al.*, 2007; Chung and Howe, 2009; Chung *et al.*, 2010; Shyu *et al.*, 2012; Thireault *et al.*, 2015). In contrast, signal or multiple mutations of *JAZ1*, *JAZ3*, *JAZ4*, *JAZ8*, *JAZ9*, *JAZ10*, and *JAZ13* lead to increased jasmonate-induced root growth inhibition (Grunewald *et al.*, 2009; Demianski *et al.*, 2012; Moreno *et al.*, 2013; Thireault *et al.*, 2015; Campos *et al.*, 2016; Guo *et al.*, 2018b). Moreover, the repressor activities of JAZ proteins are enhanced by the recruitment of the general co-repressor TOPLESS (TPL) by the adapter protein NOVEL INTERACTOR OF JAZ (NINJA). Both NINJA and TPL negatively regulate jasmonate-mediated root growth inhibition (Pauwels *et al.*, 2010). The SUMO deconjugating proteases OVERLY TOLERANT TO SALT1 (OTS1) and OTS2 repress jasmonate-modulated root growth by regulating JAZ protein SUMOylation and stability (Srivastava *et al.*, 2018). The JAZ proteins interact with ETHYLENE RESPONSE FACTOR109 (ERF109) to directly inhibit its activity during the jasmonate-mediated wound signaling that enhances root regeneration (Zhang *et al.*, 2019).

### Basic helix–loop–helix transcription factors are involved in jasmonate-induced root growth inhibition

The JAZ repressors negatively regulate jasmonate-mediated root growth inhibition mainly by interacting with and inhibiting basic helix–loop–helix (bHLH) transcription factors (Fernández-Calvo *et al.*, 2011; Song *et al.*, 2013; Fonseca *et al.*, 2014). To date, two subclades of bHLH transcription factors have been confirmed to affect the inhibition of Arabidopsis root growth by jasmonate. The most thoroughly characterized bHLH transcription factor, which is in subclade IIIe, positively affects jasmonate-mediated root growth inhibition (Boter *et al.*, 2004; Lorenzo *et al.*, 2004; Dombrecht *et al.*, 2007; Chen *et al.*, 2011; Fernández-Calvo *et al.*, 2011; Kazan and Manners, 2013). The characterized bHLH transcription factors in subclade IIId negatively affect jasmonate-related responses (Nakata *et al.*, 2013; Sasaki-Sekimoto *et al.*, 2013; Song *et al.*, 2013; Fonseca *et al.*, 2014).

**Fig. 2.**
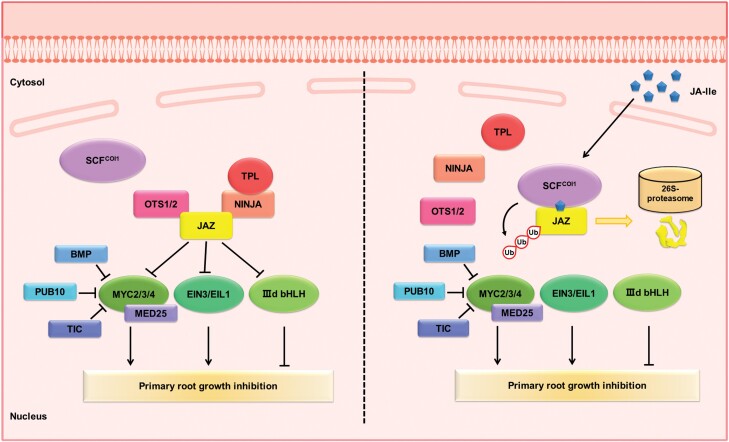
Jasmonate has an inhibitory effect on primary root growth. Jasmonate (jasmonoyl isoleucine, JA-Ile) is perceived by the F-box protein COI1, which binds to ASK1/ASK2, Cullin1, and Rbx1 to form the SCF^COI1^ complex. After jasmonate is perceived, the SCF^COI1^ complex recruits JAZ proteins, which are crucial repressors of jasmonate signaling, for subsequent degradation via the 26S proteasome pathway. The degradation of JAZ proteins alleviates their suppressive effect on transcription factors (MYC2, EIN3, IIId bHLH, and DELLA) to modulate root growth inhibition. To attenuate the inhibitory effect of jasmonate on root growth, JAZ proteins recruit a co-repressor (e.g. NINJA–TPL and OTS1), to repress downstream transcription factors. MYC2 acts a crucial role in modulating the inhibitory effect of jasmonate on root growth by interacting with other proteins (BMP, PUB, and TIC) that affects its stability and/or activity.

A previous study revealed that MYC2 and its homologs MYC3 and MYC4 are bHLH subclade IIIe transcription factors that positively regulate jasmonate responses (Fernández-Calvo *et al.*, 2011) (Fig. 2). The MYC2 transcription factor is the most prominent positive regulator because it is involved in most jasmonate-related processes, including root growth inhibition (Boter *et al.*, 2004; Lorenzo *et al.*, 2004; Dombrecht *et al.*, 2007; Chen *et al.*, 2011; Kazan and Manners, 2013). Compared with the wild-type control, the *myc2* mutant is more insensitive to jasmonate, resulting in less jasmonate-dependent root growth inhibition. Conversely, the overexpression of *MYC2* leads to increased sensitivity to jasmonate. Moreover, a comparison with a single mutant revealed an obvious decrease in jasmonate-dependent primary root growth inhibition in the *myc2 myc3 myc4* triple mutant, reflecting the functional redundancy of MYC2, MYC3, and MYC4 during the regulation of the inhibition of root growth by jasmonate (Fernández-Calvo *et al.*, 2011; Schweizer *et al.*, 2013; Gasperini *et al.*, 2015; Wang *et al.*, 2017; Wang *et al.*, 2019; Liu *et al.*, 2019).

Chen *et al.* (2011) demonstrated that MYC2 directly represses the expression of *PLETHORA* (*PLT1*) and *PLT2*, which encode key regulators of root stem cell niche maintenance, during jasmonate-mediated root growth inhibition. Furthermore, interactions with other proteins affect the stability of MYC2 and its ability to regulate jasmonate-dependent primary root growth inhibition. For example, the E3 ubiquitin ligase PLANT U-BOX PROTEIN10 (PUB10) and BTB/POZ-MATH (BPM) interact with MYC2, resulting in ubiquitination and the subsequent degradation via the 26S proteasome pathway (Jung *et al.*, 2015; Chico *et al.*, 2020). Jeong *et al.* (2017) demonstrated that polyubiquitinated MYC2 can be deubiquitinated by UBIQUITIN-PROTEASE (UBP) 12 and UBP13. The overexpression of *UBP12* or *UBP13* leads to the accumulation of MYC2 and jasmonate hypersensitivity during jasmonate-mediated root growth inhibition, whereas mutations of these genes (e.g. in the *ubp12* and *ubp13* mutants) have the opposite effects. Other studies confirmed MYC2 interacts with MEDIATOR25 (MED25), which is a subunit of a mediator complex, to activate jasmonate-modulated physiological responses (Cevik *et al.*, 2012; Chen *et al.*, 2012; Wang *et al.*, 2019) (Fig. 2).

The counterpart of the MYC subclade is bHLH subclade IIId comprising bHLH3, bHLH13, bHLH14, and bHLH17, which have repressive effects on jasmonate signaling (Nakata *et al.*, 2013; Sasaki-Sekimoto *et al.*, 2013; Song *et al.*, 2013; Fonseca *et al.*, 2014). Additionally, bHLH3, bHLH13, bHLH14, and bHLH17 have highly similar amino acid sequences and function redundantly to regulate the effects of jasmonate on root growth. An earlier investigation indicated root growth inhibition by jasmonate is much more severe in the quadruple mutant *bhlh3 bhlh13 bhlh14 bhlh17* than in the wild-type control (Song *et al.*, 2013). The overexpression of *bHLH13* and *bHLH17* attenuates jasmonate-mediated root growth inhibition (Nakata *et al.*, 2013; Song *et al.*, 2013; Fonseca *et al.*, 2014) (Fig. 2). In summary, these studies demonstrated the importance of jasmonate signaling for primary root growth in Arabidopsis. During jasmonate-induced root growth inhibition, the core components of the jasmonate signaling pathway are subject to many layers of regulation in jasmonate-induced root growth inhibition, which increase the complexity and improve the stability of jasmonate responses. Clarifying the associated spatial control circuits is critical for characterizing the broader role of jasmonate in governing root growth or other physiological processes.

### The crosstalk between the jasmonate pathway and the pathways of other phytohormones modulates root growth

Multiple recent studies elucidated the molecular basis of jasmonate-induced root growth inhibition by determining the effects of other phytohormone pathways. For example, the crosstalk between the jasmonate and ethylene (ET) pathways influences root growth. ETHYLENE INSENSITIVE 3 (EIN3) and EIN3-LIKE1 (EIL1), which are essential positive regulators of ET signaling, induce jasmonate-dependent primary root growth (Guo and Ecker, 2003; Zhu *et al.*, 2011). Genetic studies suggested that the inhibitory effects of jasmonate on root elongation are weakened in the *ein3 eil1* mutant. In contrast, transgenic plants overexpressing *EIN3* or *EIL1* are hypersensitive to jasmonate, resulting in increased root growth inhibition (Zhu *et al.*, 2011). Subsequent analysis of the associated mechanism revealed that jasmonate enhances the EIN3/EIL1 functions by removing the interacting JAZ proteins.

Gibberellins (GAs) are diterpene phytohormones that regulate plant growth and various developmental processes. The DELLA protein family includes core components of the GA signaling pathway that negatively regulate GA-modulated physiological processes (Peng *et al.*, 1997; Silverstone *et al.*, 2001; Lee *et al.*, 2002; Tyler *et al.*, 2004). Hou *et al.* (2010) reported that DELLA proteins prevent JAZ proteins from interacting with MYC2, thereby enhancing the ability of MYC2 to regulate the expression of its target genes. In plants, an increase in the GA concentration triggers the degradation of DELLA proteins, enabling JAZ proteins to interact with MYC2 and suppress MYC2-dependent jasmonate signaling. The E3 RING ligase KEEP ON GOING (KEG), which negatively regulates abscisic acid signaling via its suppressive effects on the ABSCISIC ACID INSENSITIVE5 transcription factor, directly interacts with and partially inhibits the degradation of JAZ12 during jasmonate-mediated root growth inhibition (Pauwels *et al.*, 2015). An exogenous brassinosteroid (BR) treatment can attenuate the inhibitory effects of jasmonate on root growth. Moreover, disruption of DWARF4 (DWAF4), which is a key enzyme for BR biosynthesis, renders plants hypersensitive to jasmonate during jasmonate-mediated root growth inhibition (Kim *et al.*, 2013).

### The crosstalk between the jasmonate pathway and other signaling pathways also modulates root growth

The circadian clock is an endogenous pacemaker that maintains rhythms over a 24-h period, even in the absence of the entrainment provided by daily cycles (Greenham and McClung, 2015; Takahashi, 2017). TIME FOR COFFEE (TIC) is a circadian clock regulator that maintains the circadian rhythm period and amplitude (Hall *et al.*, 2003; Ding *et al.*, 2007). Previous research indicated TIC can interact with MYC2 and repress jasmonate-mediated root growth inhibition in a MYC2-dependent manner (Shin *et al.*, 2012). A change in the red:far red light (R:FR) ratio is perceived by phytochrome photoreceptors that reversibly switch between the active (Pfr) and inactive (Pr) forms (Chen and Chory, 2011; Leivar and Quail, 2011; Burgie and Vierstra, 2014; Xu *et al.*, 2015). Under low R:FR conditions, phytochrome A (phyA) signaling limits the shade-avoidance response to enhance plant survival. Robson *et al.* (2010) demonstrated that phyA is required for jasmonate-mediated root growth inhibition. The *phyA* mutant exhibits significantly less jasmonate-induced root growth inhibition than the wild-type control.

### The crosstalk between the jasmonate and environmental stresses modulates root growth

In the natural environment, plants often endure a variety of biotic and abiotic stresses.

Jasmonate plays an important role in enhancing plant environmental adaptations by maintaining a balance between plant growth and stress tolerance. For example, jasmonate-responsive genes were up-regulated in a COI1-dependent manner upon a salt treatment in the roots. Moreover, salt stress triggers jasmonate-signaled suppression of cell elongation in the root elongation zone (Valenzuela *et al.*, 2016). In rice, OsJAZ9 interacts with OsbHLH062 to regulate ion homeostasis and improve salt tolerance (Wu *et al.*, 2015). Similarly, jasmonate enhances plant salt stress tolerance in *Lycopersicon esculentum* and *Solanum tuberosum* (Pedranzani *et al.*, 2003; De Domenico *et al.*, 2019). Boron (B) is a micronutrient essential for all phylogenetic kingdoms. In Arabidopsis, B deficiency-induced changes mainly include remodeling the root morphology, damaged xylem vessel inhibition of leaf expansion, and loss of apical dominance (Tanaka and Fujiwara, 2008; Oiwa *et al.*, 2013). B deficiency also stimulates jasmonate-induced root growth inhibition (Huang *et al.*, 2021). Adventitious roots are initiated from hypocotyls or wounded organs during regeneration in Arabidopsis (Bellini *et al.*, 2014; Xu, 2018). Jasmonate, a crucial wound-induced hormone, serves as a critical regulator of adventitious rooting from detached leaves. Zhang *et al.* (2019) revealed that, in response to wounding, jasmonate accumulates in detached leaves, increasing auxin level and promoting adventitious rooting formation.

## Jasmonate-induced root hair elongation

Root hairs are tubular polarized extensions formed by specialized cells at the root surface. They substantially increase the root surface area and facilitate the acquisition of nutrients and environmental interactions, while also increasing stress resistance and root anchorage (Peterson and Farquhar, 1996; Bibikova and Gilroy, 2003; Datta *et al.*, 2011; Vissenberg *et al.*, 2020). Root epidermal cell fates are determined in a position-dependent manner. Hair cells occur above the junction between two cortical cells, whereas non-hair cells occur above only one cortical cell (Duckett *et al.*, 1994; Galway *et al.*, 1994; Ishida *et al.*, 2008; Schiefelbein *et al.*, 2009). A group of transcription factors influencing root hair cell fate has been identified in Arabidopsis. Of these transcription factors, the R2R3-type MYB transcription factor WEREWOLF (WER), the bHLH-type transcription factor GLABLA3 (GL3) or its homolog ENHANCER OF GLABLA3 (EGL3) (Bernhardt *et al.*, 2003), and the WD repeat protein TRANSPARENT TESTA GLABLA1 (TTG1) form a protein complex that upregulates the expression of *GLABLA2* (*GL2*), which is crucial for maintaining the non-hair cell fate and repressing root hair development (Galway *et al.*, 1994; Rerie *et al.*, 1994; Di Cristina *et al.*, 1996; Masucci and Schiefelbein, 1996; Lee and Schiefelbein 1999; Bernhardt *et al*., 2003, 2005; Zhang *et al.*, 2003; Ryu *et al.*, 2005; Kang *et al.*, 2009; Schiefelbein *et al.*, 2009; S-K. Song *et al.*, 2011). Additionally, GL2 maintains the non-hair cell fate mainly by repressing the functions of bHLH transcription factors that positively regulate root hair initiation and elongation (Bruex *et al.*, 2012; Pires *et al.*, 2013; Lin *et al.*, 2015). These transcription factors include ROOT HAIR DEFECTIVE 6 (RHD6), which is a class I member of the bHLH group VIII subfamily that plays a crucial role in promoting hair cell development (Masucci and Schiefelbein, 1994; Menand *et al.*, 2007; Bruex *et al.*, 2012). Moreover, RHD6 and its closest homolog, RHD6-LIKE 1 (RSL1), have partially redundant functions related to the induction of root hair growth (Menand *et al.*, 2007). Both RHD6 and RSL1 positively regulate RSL2, RSL3, RSL4, and RSL5 (i.e. class II members of the group VIII subfamily), which also positively modulate root hair growth (Yi *et al.*, 2010; Pires *et al.*, 2013).

### Jasmonate-induced root hair development

**Fig. 3.**
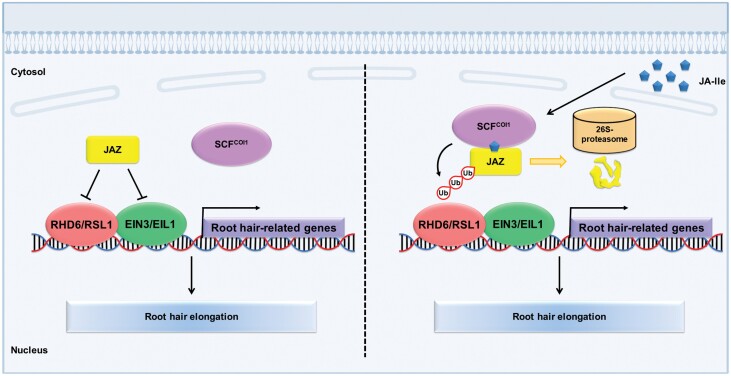
The interaction of jasmonate and ethylene pathways stimulates root hair growth. When jasmonate (jasmonoyl isoleucine, JA-IIe) is perceived, JAZ proteins are degradated, and then the JAZ-targeted transcription factors (RHD6/RSL1 and EIN3/EIL1) are released to coordinately stimulate the expression of genes essential for root hair growth.

Previous studies determined that jasmonate promotes root hair formation in Arabidopsis (Zhu *et al.*, 2006; Zhu *et al.*, 2011). However, the molecular mechanisms underlying the regulation of root hair development by jasmonate remain unknown. In a recent study, we investigated the molecular mechanisms mediating the positive effects of jasmonate on root hair growth. We found that the application of exogenous jasmonate significantly induces root hair elongation, but blocking COI1/JAZ-mediated endogenous jasmonate signaling results in defective root hairs. Jasmonate upregulates the expression of several genes encoding root hair-associated bHLH transcription factors and their downstream targets. A mechanistic investigation revealed that JAZ proteins physically interact with RHD6 and RSL1. The JAZ proteins repress the transcriptional activation of RHD6 and interfere with the interaction between RHD6 and RSL1. Most importantly, jasmonate activates root hair elongation in an RHD6/RSL1-dependent manner. Thus, we revealed a key protein complex consisting of JAZ and RHD6/RSL1 that controls root hair elongation (Han *et al.*, 2020) (Fig. 3).

### The crosstalk between jasmonate and ethylene pathways modulates root hair growth

Earlier research clarified the interactive regulatory effects of jasmonate and ET on root hair development (Zhu *et al.*, 2011). Jasmonate biosynthesis inhibitors suppress ET-induced root hair formation in the ET-overproducing mutant *eto1-1* (Zhu *et al.*, 2011). Moreover, EIN3 interacts with RHD6 to co-activate *RSL4* expression and promote root hair elongation (Feng *et al.*, 2017). Accordingly, EIN3 is a key component in the RHD6/RSL-mediated root hair initiation and elongation. As previously mentioned, EIN3 and its homolog EIL1 are targeted by JAZ repressors during the induction of root hair growth by jasmonate (Zhu *et al.*, 2011). Consequently, JAZ repressors modulate the RHD6 and RSL1 transcription factors to integrate jasmonate signaling and root hair growth partially through EIN3 and EIL1. Thus, in response to the jasmonate signal, JAZ proteins are recruited by SCF^COI1^ for subsequent ubiquitination and degradation (Chini *et al.*, 2007; Thines *et al.*, 2007; Yan *et al.*, 2009; Sheard *et al.*, 2010). As a result, the JAZ-targeted transcription factors (RHD6/RSL1 and EIN3/EIL1) are released to coordinately regulate the expression of genes essential for root hair growth (Zhu *et al.*, 2011; Feng *et al.*, 2017) (Fig. 3).

## Conclusions and future prospects

Jasmonate is a critical phytohormone that regulates plant development and adaptation to external conditions (Howe *et al.*, 2018). In response to an exposure to biotic or abiotic stress, jasmonate accumulates in plants, leading to root growth inhibition and/or root hair elongation. The jasmonate-mediated suppression of primary root development results in decreased root–pathogen interactions and pathogen-induced wounding, whereas the jasmonate-induced root hair elongation may lead to increased nutrient acquisition and enhanced stress resistance (Kawa, 2020; Li *et al.*, 2022). Thus, jasmonate-regulated root growth and root hair development is vital for plant development and adaptations to environmental conditions. However, the molecular basis of jasmonate-mediated root growth and root hair development is relatively uncharacterized. Although several previous studies demonstrated that MYC2 interacts with multiple proteins, including TPL, PUB, and MED25, to modulate root growth (Cevik *et al.*, 2012; Chen *et al.*, 2012; Jung *et al.*, 2015; Jeong *et al.*, 2017; Wang *et al.*, 2019; Chico *et al.*, 2020), additional transcription factors that can negatively regulate root growth may need to be identified and their potential relationships with specific proteins or hormones should be examined. Exploring the associated molecular mechanisms may provide new insights into how the jasmonate signaling pathway interacts with the pathways of exogenous signals (e.g. photoperiod, light signaling, and nitric oxide signaling pathways) during jasmonate-mediated root growth inhibition.

Plants tolerate various biotic and abiotic stresses throughout their life cycles. Previous studies have shown that jasmonate mediates multiple stress responses (Howe and Jander, 2008; Antico *et al.*, 2012; Erb *et al.*, 2012; Campos *et al.*, 2014; Zhang *et al.*, 2017). However, the molecular mechanisms underlying how jasmonate enhances the environmental adaptability of plants in extreme environments (e.g. nutrient deficiency, alkaline stress, high-temperature stress, and heavy metal toxicity) are uncharacterized. Future studies will be able to identify the detailed mechanisms of how jasmonate distinguishes between different environmental stresses to balance plant growth and stress tolerance by means of different strategies. Future studies could also clarify the molecular basis of the crosstalk between jasmonate and other phytohormones in mediating plant tolerance under unfavorable environments. Specifically, there are few studies regarding jasmonate-regulated crop development and adaptation to external conditions. Thus, it is also important to determine the crucial components that integrate jasmonate signaling in extreme environments to improve crop yield and environmental adaptability. Moreover, the critical components modulating jasmonate-induced root hair development need to be identified to elucidate the integrated effects of jasmonate and other hormones on root hair growth. Therefore, identifying and functionally annotating the components involved in phytohormone-regulated root growth and root hair elongation will further clarify plant adaptations to environmental conditions. Furthermore, the identification of novel genes involved in the jasmonate-regulated root growth and root hair formation of crops and the subsequent characterization of the functions of these genes and the underlying regulatory mechanisms will provide the theoretical basis for improving crop cultivation.
